# Incidence and economical effects of pneumonia in the older population living in French nursing homes: design and methods of the INCUR study

**DOI:** 10.1186/1471-2458-13-861

**Published:** 2013-09-17

**Authors:** Laurent Demougeot, Yves Rolland, Stéphane Gérard, Delphine Pennetier, Marilyne Duboué, Bruno Vellas, Matteo Cesari

**Affiliations:** 1 Gérontopôle, Centre Hospitalier Universitaire de Toulouse, Toulouse, France; 2 INSERM UMR 1027, Université Paul Sabatier - Toulouse III, Toulouse, France

**Keywords:** Older adults, Nursing home, Pneumonia, Physical disability, Health care

## Abstract

**Background:**

Among the most burdensome clinical conditions occurring in older persons, respiratory infections are particularly relevant. In fact, the onset of pneumonias is associated with a significant worsening of the individual’s global health status and significant increase of healthcare costs. The clinical and economical negative consequences of pneumonia may be particularly evident among the frailest groups of elders, in particular those living in nursing home. Nevertheless, specific research on incidence and economical effects of pneumonia in nursing homes residents is still scarce. In the present article, we present the rationale, the design and the methods of the “Incidence of pNeumonia and related ConseqUences in nursing home Resident (INCUR) study, specifically aimed at filling some of the gaps currently present in the field.

**Methods/design:**

INCUR is an observational longitudinal study recruiting 800 residents across 13 randomly selected nursing homes in France. Multidimensional evaluations of participants are conducted at the baseline, mid-term (at 6 months), and end of the study (at 12 months) visits in order to measure and follow-up their physical function, nutrition, cognition, depression, quality of life, and healthcare costs. Incident pneumonia as well as the onset/recurrence of other major health-related events are monitored during the study follow-up.

**Discussion:**

The INCUR study will provide valuable information about older persons living in nursing homes. Results from INCUR study may constitute the basis for the development of future preventive campaigns against pneumonia and its consequences.

## Background

Over the past years, the number and prevalence of older persons in industrialized countries have steadily grown [1]. Consistently, the number of people living in nursing home is also increased and is expected to further rise. To date, in the United States, there are about ~16,100 nursing homes with ~1.5 million residents for ~314 million inhabitants [2]. The projected national cost of nursing home for 2000 exceeded $140 billion, but may exceed $700 billion by 2030 [3]. In France, about 700,000 persons (representing 1.2% of the national population) [4] currently live in ~10 000 nursing homes [2]. Unfortunately, research in nursing homes is still scarce. It has been estimated that only 2% of studies in the geriatrics are carried out in this specific setting [4] which is not only characterized by a steady expansion, but is also particularly burdensome for the healthcare system.

The lack of research in nursing home may be easily explained by the complexity and heterogeneity of the older persons living in this setting and their limited access to study protocols [5,6]. The vast majority of nursing home residents is characterized by the most dramatic results of the pathophysiological modifications and chronic conditions experienced and accumulated during the entire life course. The complexity of nursing home residents is due to the high comorbidity [7,8], the frequent polypharmacy (especially including psychotropic drugs [9]) [10] the presence of major geriatric syndromes (e.g., falls [11]), and the general poor health status (e.g., malnutrition [12]). In particular, it cannot be ignored that the high prevalence of physical disability in this population. Fried and colleagues [13] calculated that although older persons represent only the 19% of the entire United States population, their healthcare needs demand almost half of the public health budget. Such economical estimation was largely influenced by the presence of disabling conditions. Both the increasing number of nursing home residents and the heavy burden they impose to public health expenditures make the study of this population a research priority in order to design and hopefully implement future *ad hoc* preventive and therapeutical interventions.

Any acute illness (in particular, respiratory conditions [14]) negatively affects physical function of older persons [15]. Therefore, the prevention of physical disability (the primary aim of geriatric medicine) may necessarily imply the need to consider preventive interventions targeting specific acute clinical conditions. The implementation of preventive campaigns is important for the subject (who may reduce the own risk of experiencing the disease) as well as for the healthcare system (which may significantly save resources).

It is possible that the frail older persons living in nursing homes may be specially amenable of beneficiating from preventive interventions and protocols [16]. In fact, the high risk of health-related events and complications to which this population is exposed may reduce the cost-effectiveness ratio of a preventive protocol. Interestingly, the same protocol may differently be unfeasible in community-dwelling subjects because a too large number of persons should receive the intervention in order to gain significant benefits. Moreover, a limited improvement (or even the avoidance of health loss) in a frail older person may have relevant effects for public health, given the exponential relationship between healthcare costs and physical function [13].

Although the incidence of infectious diseases has been largely decreasing in young and adult persons in Western Countries over the last century, similar trends have not been reported in older persons [17-20]. The atypical presentations of infections at advanced age (including lack of fever, behavioral changes, confusion) are responsible for diagnostic difficulties resulting in incorrect therapeutical prescriptions. For example, it has been reported that a significant proportion of antibiotics use is not justified or not adequately prescribed in nursing homes [21,22]. The erroneous use of antibiotics contributes not only to generate bacterial resistances, but also increases direct and indirect costs [21,23].

Most of the observational studies estimating the incidence and effects of infectious diseases has been performed at the community level. Limited evidence on this topic is currently available in nursing homes and long-term care facilities which generally host older persons with higher morbidity and increased mortality risk [20,24]. Moreover, as suggested by the large variations of prevalence and incidence rates available in literature (mainly due to differences in methodological approaches, intensity of surveillance, case mix, and facility administration [25]), data from these healthcare settings are far to be definitive. Just to provide few examples about the relevance of the (currently underestimated) phenomenon in nursing home: it has been calculated that the prevalence of infections (i.e., respiratory, cutaneous, and urinary) in institutionalized older persons may range from 1.6% to 32%, and the overall incidence rates vary from 1.8 to 13.5 infections per 1,000 residents-year [26]. These figures are at least 10-fold higher than those reported in community-dwelling older persons [27]. In this context, the respiratory system is one of the most frequently affected sites for infections. The incidence of nursing home-acquired pneumonia in the United States varies between 0.3 and 2.3 episodes per 1,000 resident-days [25]. In a recent paper, Ahmed and colleagues [28] explored the hospitalization events occurring among nursing home residents in the United States, finding that pneumonia was the leading primary discharge diagnosis from the hospital (6%) and a major predictor of in-hospital mortality.

The purpose of the present article is to present the design and methodological approach of the “Incidence of pNeumonia and related ConseqUences in nursing home Resident” (INCUR) study. INCUR is an observational longitudinal cohort study aimed at estimating the incidence and economical effects of pneumonia in older persons living in nursing homes (i.e., *Etablissement d’Hébergement pour Personnes Agées Dépendantes*, EHPAD) in France.

### The INCUR study

#### Hypotheses and objectives

The INCUR project is specifically aimed at retrieving data from routine clinical practice in order to estimate the incidence of pneumonia events in older persons living in a sample of French nursing homes. Moreover, INCUR is designed to estimate the clinical and economical consequences of pneumonia events by measuring the subsequent access to medical procedures and resources. More in details, the following hypotheses have driven the rationale and conception of the INCUR project:

Primary hypotheses:

a. Pneumonia is a clinical condition frequently complicating the nursing home stay of older persons;

Secondary hypotheses:

a. The onset of a pneumonia event in institutionalized older residents is associated with higher healthcare costs;

b. Incident pneumonia events are associated with an increased risk of mortality in older persons living in nursing home;

c. The onset of a pneumonia event is responsible for a significant worsening of the physical function of nursing home residents;

Tertiary hypotheses:

a. Specific variables at patient- and nursing home-level may predict the severity of the pneumonia-related functional loss;

b. Nursing home residents dying due to pneumonia produce a significantly higher burden in terms of healthcare costs compared to nursing home residents dying for other causes.

Therefore, the INCUR project will pursue the following aims:

Primary aims:

a. To estimate the incidence of pneumonia events in a sample of older nursing home persons over a period of 12 months;

Secondary aims:

a. To compare the healthcare costs of nursing home older persons experiencing a pneumonia event versus those who will not over a period of 12 months;

b. To calculate the mortality risk in a sample of older residents living in nursing home according to the presence/absence of a pneumonia event occurred during a follow-up period of 12 months;

c. To measure the physical function modifications experienced by a sample of nursing home residents according to the presence/absence of a pneumonia event occurred during a follow-up period of 12 months;

Tertiary aims:

a. To identify sociodemographic, clinical, and biological characteristics of nursing home older residents, as well as environmental and organizational factors of their own hosting institution, which are associated with a higher degree of functional loss following a pneumonia event;

b. To explore whether mortality due to pneumonia is associated with higher healthcare costs compared to death events due to non-respiratory causes in nursing home older residents.

## Methods/design

For the INCUR project, we recruited 800 residents of 13 nursing homes randomly selected in the Midi-Pyrénées region of France. In particular, nursing homes of the region were consecutively and randomly invited at participating in INCUR. After each nursing home accepted to be involved in the study, all its residents (according to their eligibility for INCUR) were assessed by the study personnel. The recruitment was conducted up to the reaching of the final sample of 800 participants according to the predefined sample size analyses (see specific paragraph). All the nursing homes were part of a national network of institutions representative of this healthcare in France [4]. To be part of the project, the director, the coordinating physician, and the responsible for the nursing of each institution signed a formal agreement and letter of support.

The main inclusion and exclusion criteria were the constituted by the score obtained by the participant at the *Autonomie Gérontologie - Groupes Iso-Ressources* (AGGIR) scale (Table 1). This scale allows to homogeneously generate six *Groupes Iso-Ressources* (GIR) at the French national level. The resulting GIR expresses the global functional capacity of the person and drives decisions about the provision of social support from public healthcare system. Subjects with severe disability are excluded from INCUR on the basis of the worst GIR group (i.e., 1). The reason for such exclusion resides in the impossibility to determine in these patients the weight of functional loss due to a pneumonia event (primary aim d). Similarly, residents with no sign of functional impairment at the AGGIR scale (that is having the maximum GIR score of 6) are excluded due to the incapacity to perceive possible functional improvement over the follow-up.

**Table 1:** Inclusion and exclusion criteria of the INCUR study

*Inclusion criteria*	*-* Residents aged 60 years and older
	*- Groupes Iso-Ressources* (GIR) ranging between 2 (included) and 5 (included). This is the French administrative tool used to rate the ability of the person to be independent; it ranges from 6 (fully independent) to 1 (fully dependent, bed-ridden)
*Exclusion criteria*	- Residents living in the participating nursing homes for less than 30 days since the baseline study visit
	- Refusal of the patient and/or the family to participate at the INCUR project

**Figure 1:**
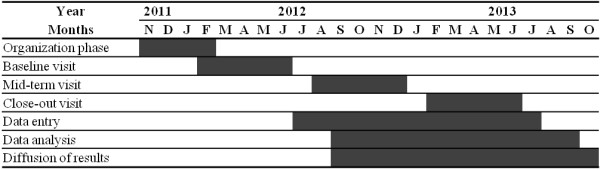
The timeline of the INCUR project.

Each eligible participant will be followed-up for 12 months with a total of three clinical assessments: at baseline, 6 months (mid-term visit), and 12 months (close-out visit). The timeline of the INCUR project is depicted in Figure 1. Recruitment of participants and baseline visits began in February 2012 and ended in July 2012, after a preliminary organization phase (for coordinating activities of researcher with the participating nursing homes). The mid-term visit of participants (after 6 months of follow-up) is currently ongoing.

The INCUR visits are conducted by research staff specifically trained at the Gérontopôle of Toulouse. The evaluations will be conducted using multiple scales, questionnaires and tests based on the Geriatric Minimum Data Set (GMDS) [29], thus allowing a comprehensive assessment of participants. The GMDS is a package of assessment tools designed by a multidisciplinary task force group (i.e., the Gerontonet) [30], aimed at evaluating the different areas of the person’s health, including physical function, nutrition, cognition, depression, quality of life. As shown in Table 2, these include: ADL [31], Instrumental ADL (IADL) [32], European Quality of Life (EuroQoL) instrument [33], frailty phenotype proposed by Fried and colleagues [34], Abbreviated Mental Test [35], 15-item Geriatric Depression Scale [36], Mini Nutritional Assessment [37]. The GMDS instrument was originally conceived to promote the translation of routinely collected clinical parameters into research data. Therefore, consistently with the descriptive nature of the INCUR project, the items collected will be primarily retrieved from the medical charts of the participants (if present). Missing data will be completed by specific evaluations of the INCUR study staff.

**Table 2:** Main areas investigated and related instruments adopted in the INCUR project

**Area**	**INCUR assessment**
Sociodemographics	
Body composition	Anthropometric measures
Anamnesis/Comorbidity	Current diseases
Medications	
Vaccinations	Influenza and pneumococcal vaccine
Cognition	Abbreviated Mental Test
	Geriatrics Depression Scale
Quality of life	Euro-QoL 5D
Pain	Pain Visuo-Analogic Scale
Functional Status	Activities of Daily Living
	Instrumental Activities of Daily Living
	Short Physical Performance Battery
	Hand grip strength
Nutrition	Mini Nutritional Assessment
Healthcare cost	Data from the *Caisse Primaire d’Assurance Maladie*
Follow-up	Intercurrent diseases
	Therapy modifications
	Hospitalization events
	Death events and causes of death

The same standardized multidimensional assessment performed at the baseline will then be repeated at the 6- and 12-month follow-up visits. Additionally, the follow-up visits will be specifically focused at identifying the onset of major health-related events (in particular pneumonia) occurred during the previous 6 months of follow-up. In particular, the study staff will retrieve information about major intercurrent illnesses, emergency room admissions, hospitalizations, and death events (including causes) from patient's medical records and charts, the nursing home personnel, and the proxies of the participant. Moreover, in between the follow-up visits, regular contacts between the study staff and the nursing homes personnel will be maintained in order to facilitate and improve the identification of the health-related events experienced by the INCUR participants.

To design and develop the INCUR study, the principles of the Declaration of Helsinki have been followed and ethical standards complied. The Ethics Committee of the Centre Hospitalier Universitaire de Toulouse and the Consultative Committee for the Treatment of Research Information on Health (CNIL) approved the entire study protocol. The Ethics Committee waived the need for written informed consent from the participants given the epidemiological nature of the research within the domain of current clinical practices. Therefore, participants are not required to provide a written informed to the study, but all participants receive written information about the ongoing research (including its objectives and procedures) by the study investigators. Residents are then free to exclude their data from the collection or analysis at any time. In case of residents with cognitive impairment or unable to understand the research protocol, proxies are informed.

### Pneumonia

From a clinical standpoint of view, it is difficult to obtain a clear and definitive diagnosis of pneumonia in the nursing homes population beacause of the multitude of comorbidities (e.g., heart failure, chronic obstructive pulmonary disease, pulmonary fibrosis) often altering the manifestation of the respiratory infection. For example, cough is often absent (even in more than 50% of the cases), pulmonary crackles are rare, and the only reliable criterion remains the increased respiratory rate. Such difficulties combined with the limited access to complementary biological or imaging exams lead to unreliable estimates for the diagnosis of pneumonia in the nursing homes setting. To improve the diagnostic definition of pneumonia, the *Observatoire du Risque Infectieux en Gériatrie* (ORIG) [38] recently published specific criteria refining those previously proposed by McGeer and colleagues [39]. The ORIG criteria described by Rothan-Tondeur and colleagues [38] are specifically adapted for use in the nursing homes, taking into account the diagnostic difficulties characterizing this clinical setting. Thus, the ORIG definition of a pneumonia event mirrors the one based on the McGeer and colleagues’ algorithm [39], but replace the most invasive and complex endoscopic and microbiological criteria with the physician’s clinical judgement.

In the INCUR study, consistently with the ORIG proposition, we will identify pneumonia events on the basis of the following two criteria:

1. Presence of at least two of the following signs/symptoms:

a. Worsening or onset of cough, purulent sputum, or specific signs at the auscultation,

a. Fever (≥38°C),

a. Thoracic pain,

a. High respiratory rate (≥25 breaths per minute),

a. Mental confusion or worsening of physical disability, and

2. Clinical evidence documented by a physician of crackles at the thoracic auscultation.

It has been reported that the probable cases identified using the ORIG definition are about 3.5-fold higher than the definitive cases of pneumonia identified using the McGeer and colleagues criteria [39]. Surely, this latter definition is more accurate and completely based on objective data. However, its difficult clinical implementation (especially in the nursing homes) and the large difference in the identified cases of pneumonia (compared to the ORIG definition) may be responsible for a likely underestimation of the phenomenon and an inadequate capacity to be adapted to such healthcare setting.

### Physical function measures

Physical function modifications will be determined by repeated evaluations of several specific scales and questionnaires. In particular, we will use:

1. ADL scale [31]; The number of impaired ADLs was calculated considering the following six tasks: transferring from bed to chair, walking across a room, eating, bathing, using the toilet and personal hygiene.

2. IADL scale [32]; The assessment of impaired IADLs was based on the evaluation of the following four tasks: use of the telephone, use of transportations, managing medications and managing finances.

3. Short Physical Performance Battery (SPPB) [40,41]. The SPPB is a physical performance test composed by three timed subtasks: 4-meter usual gait speed, balance, and chair stand tests. Timed results from each test are scored from 0 (worst performers) to 4 (best performers) according to validated and standardized cut-points [41]. Participants unable to perform a specific SPPB subtest will result with a score of 0 (or the 99th percentile) for that part of the instrument. SPPB has shown to be associated with subclinical (e.g., inflammation, vitamin D, antioxidant status) [42,43] and clinical (e.g., comorbidity, disability, hospitalization, institutionalization, mortality) [44,45] in older persons;

4. Hand grip strength. It will be measured using a hand-held dynamometer (model Jamar). Participants will be asked to perform the task twice with each hand. The average of the best results obtained at each side will be used for the analyses. Hand grip strength has shown to be predictive of health-related events (including disability and mortality) [44,46-48];

5. *Groupes Iso-Ressources* (GIR). According to results of this scale, a special allowance (i.e., *Allocation Personalisée d'Autonomie*, APA) is allocated by the Conseil Général to functionally impaired subjects (scoring 4 or lower). Therefore, its significance is beyond a pure evaluation of physical function, but represents an easy and early indicator of the healthcare cost of an older person.

### Healthcare costs

To address the second aim of the present project related to the healthcare costs of pneumonia events in older persons living in nursing homes, the INCUR project will obtain specific data from the *Caisse Primaire d’Assurance Maladie* (i.e., the French national healthcare insurance system). In particular, the study coordinators will receive the direct costs that the healthcare system will sustain for each INCUR participant during the study period. Such figures will include total and detailed expenditures (including performed medical procedures, medications consumption, access to specific healthcare services…). As mentioned, specific parameters acting as surrogated measures of healthcare expenditures will also be directly retrieved during the INCUR clinical visits from the participants’ medical records and charts by the INCUR personnel. In particular, we will evaluate the following parameters: number and length of hospitalization events, number of major medical procedures (including admission and length of stay in Intensive Care Unit, Orotracheal intubation, mechanical ventilation), and transition to worse GIR classes. Although these (mainly clinical) parameters may not provide the exact estimation of the costs sustained by the healthcare system for the patient, they still represent reliable markers of the economical weight of the case. Moreover, since all these procedures and events have direct and negative influences on the older patient’s quality of life and functional status, their significance goes well beyond a mere financial evaluation. The collaboration with the CNAM will provide us a detailed estimation of healthcare costs charged to the public healthcare system for the management of INCUR participants. Nevertheless, the main limitation of INCUR in this context is represented by the impossibility to detect and measure costs directly sustained by the participant (and his/her family) and the impact of caregivers’ time on societal aspects.

### Sample size analyses

Mehr and colleagues [25] estimated that the incidence of nursing home-acquired pneumonia in the United States may range between 0.3 to 2.3 episodes per 1,000 resident-days. In the present study, we expect to enroll 800 EHPAD residents and follow them over a 12-month period (i.e., a total of 292,000 resident-days). Considering the attrition due to mortality (i.e., 20% per year in this setting^4^), we may expect between 70.1 and 537.3 cases according to the Meher and colleagues’ data [25]. More recently, Rothan-Tondeur and colleagues [38] reported 311 infections in a sample of 2,140 nursing home residents over a period of 28 days (from January 24 to February 20, 2005). The study was conducted defining pneumonia according to the same criteria we will implement in INCUR. Authors showed that pneumonia represented the 7% of all the cases of infections (i.e., 22 events). Translating these figures over a 365-day period (as proposed in the INCUR project) and sample size we plan to recruit (i.e., 800 participants), we obtain 107.2 pneumonia events. It might happen that attrition due to mortality (i.e., 20% per year^4^) and the spring-summer seasons (during which the pneumonia events might be fewer; i.e., additional 40% over 6 months) may further reduce such estimation. However, we believe that, even in the worst case scenario (i.e., 68.6 cases of pneumonia over one year, consistent with the minimum estimation obtained from analyses performed on the Meher and colleagues’ data [25]), it will be possible the evaluation of a sufficient number of events to successfully accomplish the INCUR study aims. The recruited sample size of 800 participants produces a two-sided 98.2% or 95.2% confidence interval with a width equal to 0,050 when the incidence of pneumonia are 10% or 15%, respectively. Such incidence rates are overestimating the outcome of interest compared to the previous study by Rothan-Tondeur et al [38]. (7%); thus, INCUR study is adequately powered to accurately estimate the incidence of pneumonia with the sample of 800 participants.

### Analysis plan

All the analyses will consider an alpha value threshold equal to 5% as determining the statistical significance.

Primary aims:

a. Taking into account the 12-month follow-up of the study participants, we will detect all the participants experiencing a new pneumonia event. The ratio between the number of participants experiencing one or more events and the number of participants constituing the overall sample will provide the incidence of pneumonia in the sample population. Results will be presented as pneumonia cases per 1,000 resident-days, and as pneumonia events over one year. Stratified analyses will be performed according to age groups, gender, and pre-existing pulmonary disease.

Secondary aims:

a. Primary independent variable of interest for the present analyses will be the total expenditure (in Euros) that participants will cost to the healthcare system over the follow-up year. Additional secondary analyses will also consider specific aspects of the direct healthcare costs of INCUR participants such as expenses due to hospitalizations or physical therapy sessions. All these analyses will be stratified and compared according to the presence/absence of pneumonia events.

Unadjusted and adjusted analyses of covariance, logistic regression models, and multinominal logistic regression models will be performed to evaluate the means and risks of the outcome variables predicted by the absence/presence of pneumonia events occurred during the follow-up (dicothomous independent variable of interest).

b. Participating patients will be divided in two groups, according to the presence/absence of a pneumonia event experienced during the follow-up period. Then, the risk of death will be calculated and compared between the two groups. Kaplan-Meier survival curves will be provided according to pneumonia events. Moreover, adjusted Cox proportional hazard analyses taking into account age, gender, pre-existing pulmonary disease, and other possible confounders will be performed to estimate the risk of dying in patients who experienced a pneumonia versus those who did not. Follow-up will be censored at the date of death for patients dying during the follow-up, or to the last contact date for those who did not.

c. When a pneumonia event will occur, physical function information collected after its onset will be compared to those previously recorded. The difference in physical function measured before and after the pneumonia event will be adjusted for the days between the event onset and the following physical function assessment. This analytical approach will allow to take into account the negative influence that the presence of a (sub)acute phase of the respiratory process may exert on physical function if the event will occur close to the evaluation. Mixed effects analysis of covariance models will also be performed to evaluate physical function modifications over time between patients who experienced a pneumonia event versus those who did not. If statistical power will be sufficient, stratified analyses will also be performed according to the severity of the pneumonia event (e.g., events requiring hospitalization versus those who did not).

For the present analyses, the sum of the results from the three SPPB categorized tests (ranging from 0 [worst physical performance] to 12 [best physical performance]) as well as the crude timed results obtained from each single assessment (in seconds) will be used for the analyses. This will allow to evaluate whether the pneumonia event will differently affect specific components of physical performance (i.e., balance, muscle strength, or gait/coordination).

Tertiary aims:

a. Chi-square and t-test analyses will be performed to describe the study population for main sociodemographic, behavioral, and clinical characteristics according to the presence/absence of a pneumonia event occurred during the INCUR project follow-up. Mann-Whitney U statistics will be used for non-normally distributed continuous variables. Normalization through log-transformation will be used for non-normally distributed study variables. Subsequent unadjusted and adjusted Cox proportional hazard models will be analyzed to identify patients’ characteristics that are able to significantly predict pneumonia events (dependent variable of interest). Among the covariates, age, gender, race, and preexisting respiratory conditions will always be considered as potential confounders. Age, gender, and race interactions will be tested for the studied relationships by adding specific interaction terms into the models. If possible, stratified analyses will be performed whereas significant interactions will be obtained.

A similar analytical approach will be used to evaluate specific characteristics of the EHPADs as significant determinants of pneumonia events in patients. EHPADs and patients’ characteristics might be combined in secondary analyses to evaluate the additive value of specific parameters towards the onset of pneumonia events.

b. We will perform restricted analyses in participants who will die during the follow-up (expected mortality about 20% over 12 months, that is 160 fatal events in our sample population). This subsample of participants will be divided according to the cause of death (i.e., pneumoniarelated versus other causes [expected number of cases 11.2 versus 158.8 according to Rothan- Tondeur et al [38]). Chi-square and t-test analyses will be performed to verify whether significant differences between patients dying because of a pneumonia event versus those dying for other causes exist for the following healthcare parameters: - Total expenditure (in Euros) that participants will cost to the healthcare system over the follow-up period. This datum will be obtained by the Gerontopôle from the *Caisse Primaire d’Assurance Maladie*, which has allowed the specific and exclusive release of it to the CHUToulouse. Additional analyses will also consider specific aspects of the direct healthcare costs of INCUR participants such as expenses due to hospitalizations or physical therapy sessions.

Mann-Whitney U statistics will be used for non-normally distributed continuous variables. Normalization through log-transformation will be used for non-normally distributed study variables. These analyses will consider the entire follow-up period available. Secondary analyses will also be restricted to consider the last month preceding the fatal event. In this way, we will be able to undestand whether the hypothesized higher healthcare costs of pneumonia events leading to death are distributed over time, or compressed in the very last period of life. If the statistical power will be sufficient, additional adjusted logistic and linear regression models will be performed taking into account potential confounders.

### Quality control

The quality and eventual acceptance of all studies depend on issues such as: maintaining randomization integrity, accurately assessing participant eligibility, recording dropouts and adherence, measuring outcome variables without bias, preventing premature release of results, monitoring and assessing protocol adherence, and avoiding biases in the analysis of the results. Quality control procedures are devised to monitor screening, data collection, follow-up, clinical measurements, collection of forms, data entry procedures, and overall scientific and leadership operations.

## Discussion

Pneumonia is the leading cause of mortality, morbidity and transfers to acute care facilities among nursing homes residents. Nursing home-acquired pneumonia absorbs a large part of economical resources of the healthcare system [49]. There is growing evidence suggesting that hospitalization events in residents with nursing home-acquired pneumonia may also not be clinically relevant, but still responsible for increased healthcare costs [50]. Unfortunately, clinical research in the nursing home population is still too limited in this field.

The extremely heterogeneous characteristics of institutionalized older persons make particularly difficult the design and conduction of specific studies and, at the same time, almost impossible to directly apply evidence from different settings. The value of preventive or therapeutic strategies commonly adopted in adults is often insufficiently assessed or adequately confirmed/validated in institutionalized older persons to allow a direct and unmodified translation of medical guidelines. This issue may (at least partially) explain the relative reduction of efficacy for several interventions (including antibiotics) in nursing homes [51].

Given the multicausal nature of disability, preventive interventions against it should target a wider spectrum of risk factors potentially inducing (or worsening) the loss of physical function and health status. Consequently, an effective program of prevention against disability goes necessarily through the counteraction of stressors (including acute conditions, such as pneumonia) capable to disrupt the already impaired homeostatic equilibrium of frail older persons. In such scenario, preventive interventions (e.g., anti-pneumococcal vaccines) may gain special relevance, even beyond their specific action at the respiratory apparatus level.

The current limited evidence coming from the nursing home setting and the modest adherence to current recommendations represent two major barriers to successful act against disability and its major consequences at advanced age. Rolland and colleagues [4] already discussed the reasons for this gap between the theoretical scientific recommendations and their practical application in the nursing home clinical routine. For example, data from a recent study reveal that while most nursing home residents (about 80%) currenty receive anti-influenza vaccination, less than one third are instead vaccinated against pneumococcus [52]. To achieve higher vaccination rates, the use of Standing Order Program (allowing for vaccination without an individual physician's order) has even been proposed as being the most effective and economically favourable strategy to implement preventive interventions [53]. However, before considering the systematic implementation of an intervention like this (especially given the size of the target population) more robust data are needed. In other words, it is important to carefully and more comprehensively study the most common conditions of nursing homes, in particular those diseases for which the efficacy of preventive and/or therapeutical interventions has already been clearly proven elsewhere. Only after having obtained data describing the disease characteristics (in terms of prevalence, incidence, consequences) and the cost-effectiveness of available preventive/therapeutical interventions, the extension of these latters may be correctly planned in the daily clinical routine of nursing homes. The INCUR project goes indeed in this direction by 1) describing an almost unknown population, 2) estimating the incidence of a frequent and severely burdening condition as pneumonia, 3) measuring the effects of such disease in terms of clinical (for the patient) and economical (for public health) consequences.

In conclusion, studies are needed to adequately describe and explore the complex frail older population living in nursing homes. The INCUR study will provide valuable information about the incidence and consequences of a frequent clinical condition (i.e., pneumonia) in this peculiar subgroup of older persons. The INCUR study will also support the conduction of sample size analyses of future trials aimed at evaluating the effects of preventive/therapeutical interventions against pneumonia in nursing homes. Main results from the INCUR study are expected for the second half of 2013.

## Competing interest

All authors declare no conflict of interest.

## Authors’ contributions

MC, YR, BV: have made substantial contributions to conception and design. LD, DP, MD: have participated to the data collection. LD, MC: wrote the manuscript. LD, YR, SG, DP, MD, BV, MC: have made substantial contributions to the final manuscript. All authors read and approved the final manuscript.

## Pre-publication history

The pre-publication history for this paper can be accessed here:

http://www.biomedcentral.com/1471-2458/13/861/prepub
